# Decoding speech from spike-based neural population recordings in secondary auditory cortex of non-human primates

**DOI:** 10.1038/s42003-019-0707-9

**Published:** 2019-12-11

**Authors:** Christopher Heelan, Jihun Lee, Ronan O’Shea, Laurie Lynch, David M. Brandman, Wilson Truccolo, Arto V. Nurmikko

**Affiliations:** 1 School of Engineering, Brown University, Providence, RI USA; 2 Connexon Systems, Providence, RI USA; 3 Department of Surgery (Neurosurgery), Dalhousie University, Halifax, Nova Scotia Canada; 4 Department of Neuroscience, Brown University, Providence, RI USA; 5 Carney Institute for Brain Science, Brown University, Providence, RI USA

**Keywords:** Neural decoding, Cortex

## Abstract

Direct electronic communication with sensory areas of the neocortex is a challenging ambition for brain-computer interfaces. Here, we report the first successful neural decoding of English words with high intelligibility from intracortical spike-based neural population activity recorded from the secondary auditory cortex of macaques. We acquired 96-channel full-broadband population recordings using intracortical microelectrode arrays in the rostral and caudal parabelt regions of the superior temporal gyrus (STG). We leveraged a new neural processing toolkit to investigate the choice of decoding algorithm, neural preprocessing, audio representation, channel count, and array location on neural decoding performance. The presented spike-based machine learning neural decoding approach may further be useful in informing future encoding strategies to deliver direct auditory percepts to the brain as specific patterns of microstimulation.

## Abstract (web summary)

Heelan, Lee et al. collect recordings from microelectrode arrays in the auditory cortex of macaques to decode English words. By systematically characterising a number of parameters for decoding algorithms, the authors show that the long short-term memory recurrent neural network (LSTM-RNN) outperforms six other decoding algorithms.

## Introduction

Electrophysiological mapping by single intracortical electrodes has provided much insight in revealing the functional neuroanatomical areas of the primate auditory cortex. Directly relevant to this work is the investigation of the role of the macaque secondary auditory cortex in processing complex sounds through intracortical microelectrode array (MEA) population recordings. Whereas the core of the secondary auditory cortex lies in the lateral sulcus, portions of the adjacent belt and parabelt lie on the superior temporal gyrus^1^ (STG). This area is accessible to chronic implantation of MEAs for large channel-count broadband recording (see “Methods” section). Prior seminal research into the STG has provided understanding of the hierarchical processing of auditory objects by producing detailed maps of the cellular level characteristics through microwire recordings^1–6^. The connection of non-human primate (NHP) research to human speech processing has been reviewed^7^. Additional techniques to acquire global maps of the NHP auditory system (via tracking metabolic pathways) include fMRI^8^ and 2-deoxyglucose (2-DG) autoradiography^9^.

Other relevant animal studies have examined the primary auditory cortex (A1) in ferrets (single neuron electrophysiology) to unmask representations underlying tonotopic maps^10,11^. This research has demonstrated encoding properties based on spectrotemporal receptive fields (STRFs) of the primary sensory neurons in A1. Further, studies in marmosets have yielded key insights into neural representation and auditory processing by mapping neuronal spectral response beyond A1 into the belt and parabelt regions of the secondary auditory cortex through intrinsic optical imaging^12^. Results suggest that the secondary auditory cortex contains distributed spectrotemporal representations in accordance with findings from microelectrode mapping of the macaque STG^4^. Another recent study examined feedback-dependent vocal control in marmosets and showed how the feedback-sensitive activity of auditory cortical neurons predicts compensatory vocal changes^13^. Importantly, this work also demonstrated how electrical microstimulation of the auditory cortex rapidly evokes similar changes in speech motor control for vocal production (i.e., from perception to action).

We also note relevant human research which mainly deployed intracranial electrocorticographic (ECoG) surface electrode arrays in the STG^14–22^. While surface electrodes are thought to report neural activity over large populations of cells as field potentials, relatively accurate reconstructions of human and artificial sounds have been achieved in short-term recordings of patients during clinical epilepsy assessment. The recordings generally focus on multichannel low-frequency (0–300 Hz) local field potential (LFP) activity, such as the high-gamma band (70–150 Hz), using linear and nonlinear regression models. LFP decoding has been used to reconstruct intelligible audio directly from brain activity during single-trial sound presentations^15,19^. When combined with deep learning techniques, electrophysiology data recorded by ECoG grids has been decoded to reconstruct English language words and sentences^23^. These studies provided insight into how the STG encodes more complex auditory aspects such as envelope, pitch, articulatory kinematics, spectrotemporal modulation, and phonetic content in addition to basic spectral and temporal content. One study augmented findings from ECoG studies by recording from an MEA implanted on the anterior STG of the patient where the single or multiunit activity showed selectivity to phonemes and words. This work provided evidence that the STG is involved in high-order auditory processing^24^.

**Fig. 1:**
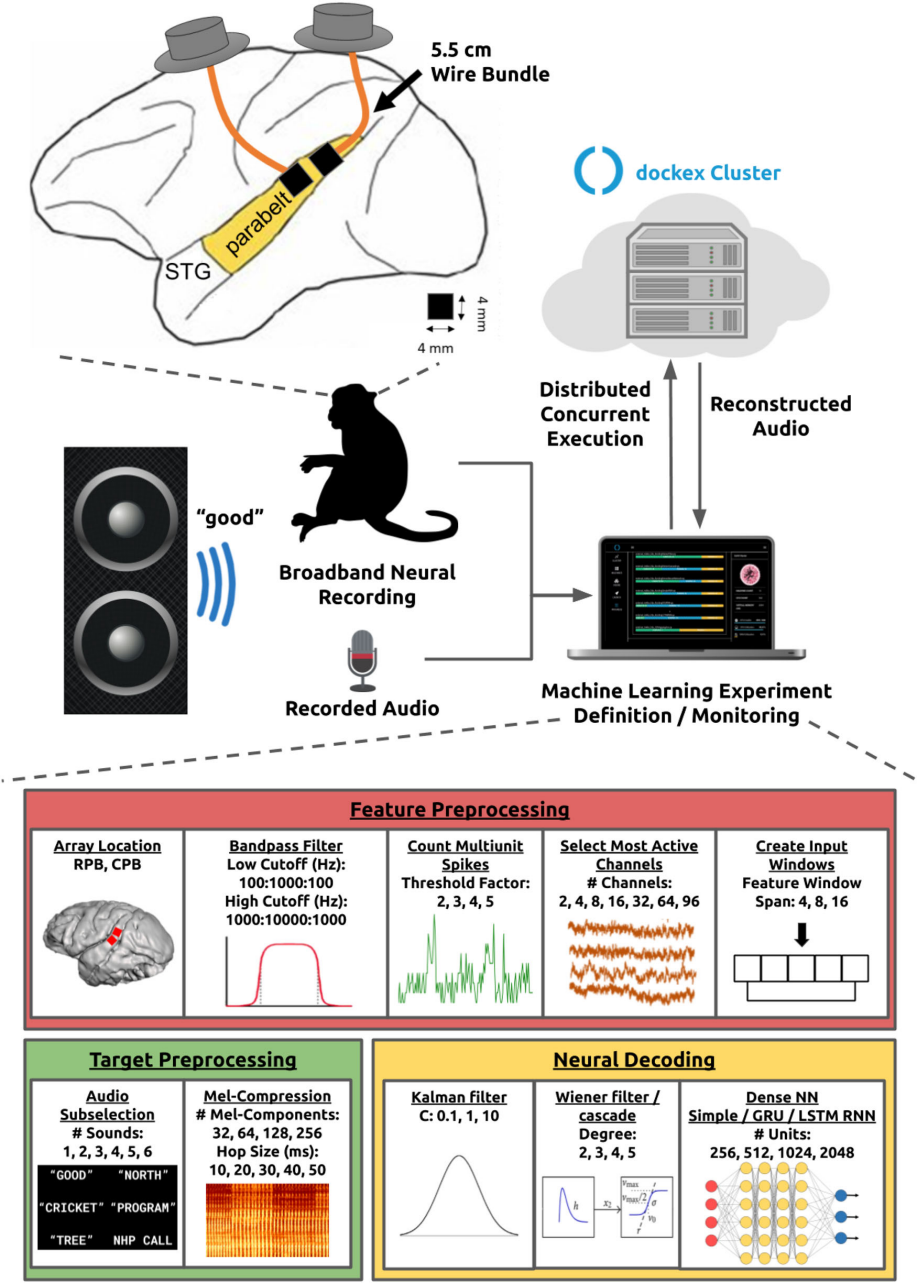
We implanted two NHPs with MEAs in the STG. We presented the subject with six recorded sounds and processed neural and audio data on a distributed cluster in the cloud.

In this paper, we investigated whether accurate low-order spectrotemporal features can be reconstructed from high spatial and temporal resolution MEA-based signals recorded in the higher-order auditory cortex. Specifically, we explored how different decoding algorithms affect reconstruction quality when availed to large channel count neural population recordings of spikes. We implanted two 96-channel intracortical MEAs in the parabelt areas of the secondary auditory cortex in the rhesus macaque model and demonstrated the successful decoding of multiunit spiking activity to reconstruct intelligible English words and macaque call audio (see Fig. 1). Further, using a novel neural signal processing toolkit^25^ (NPT), we demonstrated the effects of decoding algorithm, neural preprocessing, audio representation, channel count, and array location on decoding performance by evaluating thousands of unique neural decoding models. Through this methodology, we achieved high fidelity audio reconstructions of English words and macaque calls through the successful decoding of multiunit spiking activity.

## Results

A Supplementary Summary Movie^26^ was prepared to give the reader a concise view of this paper. We used 96-channel intracortical MEAs to wirelessly record^27,28^ broadband (30 kS/s) neural activity targeting Layer 4 of the STG in two NHPs (see “Methods” section). We played audio recordings of 5 English words and a single macaque call over a speaker in a random order. Using a microphone, we recorded the audio playback synchronously with neural data.

We performed a large-scale neural decoding grid-search to explore the effects of various factors on reconstructing audio from the subject’s neural activity. This grid-search included all steps of the neural decoding pipeline including the audio representation, neural feature extraction, feature/target preprocessing, and neural decoding algorithm. In total, we evaluated 12,779 unique decoding models. Table 1 enumerates the factors evaluated by the grid-search. Additionally, we evaluated decoder generalization by characterizing performance on a larger audio data set (17 English words) and on single trial audio samples (3 English words not included in the training set).

**Table 1:** The searched algorithm and hyperparameter space.

Neural decoders	
Kalman filter	C: 0.1, 1, 10
Wiener filter	
Wiener cascade	Degree: 2, 3, 4, 5
Dense Neural Network (NN)	# Units: 256, 512, 1024, 2048
Simple Recurrent NN (RNN)	# Units: 256, 512, 1024, 2048
Gated Recurrent Unit (GRU) RNN	# Units: 256, 512, 1024, 2048
Long Short-Term Memory (LSTM) RNN	# Units: 256, 512, 1024, 2048

Neural feature extraction	
Array Location:	rostral parabelt (RPB), caudal parabelt (CPB)
Filter Low Cutoff (Hz):	100 to 1000, increments of 100
Filter High Cutoff (Hz):	1000 to 10000, increments of 1000
Threshold Factor:	2, 3, 4, 5
# Channels:	2, 4, 8, 16, 32, 64, 96
Feature Window Span:	4, 8, 16

Audio processing	
Sounds:	“tree”, “good”, “north”, “cricket”, “program”, macaque call
# Sounds:	1, 2, 3, 4, 5, 6
# Mel-Bands:	32, 64, 128, 256
Hop Size (ms):	10, 20, 30, 40, 50

### Supplementary movies

During our analysis, we primarily used the mean Pearson correlation between the target and predicted audio mel-spectrogram bands as a performance metric for neural decoding models^15^. We present examples in a Supplementary Correlation Movie^29^ that demonstrates various reconstructions and their corresponding correlation scores. This movie aims to provide subjective context to the reader regarding the intelligibility of our experimental results. Additionally, we evaluated neural decoding models with other metrics that quantified reconstruction performance on specific audio aspects including sound envelope, pitch, loudness, and speech intelligibility (see “Other decoding algorithm performance metrics” section). We have also provided a Summary Movie that describes the presented findings^26^.

### Neural decoding algorithms

Neural decoding models regressed audio targets on neural features. We used the mel-spectrogram^30^ representation (128 bands) of the audio as target variables (one target per band) and multiunit spike counts as neural features (one feature per MEA channel) (see “Methods” section). Both the mel-spectrogram bands and multiunit spike counts were binned into 40 ms time bins, and all audio was reconstructed on a bin-by-bin basis (i.e., no averaging across bins or trials).

Data were collected from a total of 3 arrays implanted in 2 NHPs (RPB and CPB arrays in NHP-0 and an RPB array in NHP-1). Data for each array were analyzed independently (i.e. no pooling across arrays or NHPs). Unless explicitly stated, all models were trained on 5 English words using all 96 neural channels from the NHP-0 RPB array. Each array data set contained $\sim$40 repetitions of each word (words randomly interleaved) collected over multiple sessions.

To mitigate decoding model overfitting, data collected from a given array was sequentially concatenated across recording sessions, and the resulting array data sets were sequentially split into training (80%), validation (10%), and testing (10%) sets. The primary motivation for this work was to enable future active listening tasks that will consist of a limited number of English words ($\sim$3 to 5 words) and subsequent neural encoding experiments that will elicit auditory sensations through patterned microstimulation. As such, multiple presentations of all 5 words were present in the training, validation, and test sets. For an analysis of decoding performance on words not included in the training set, see “Decoding larger audio sets and single trial audio” section.

**Fig. 2:**
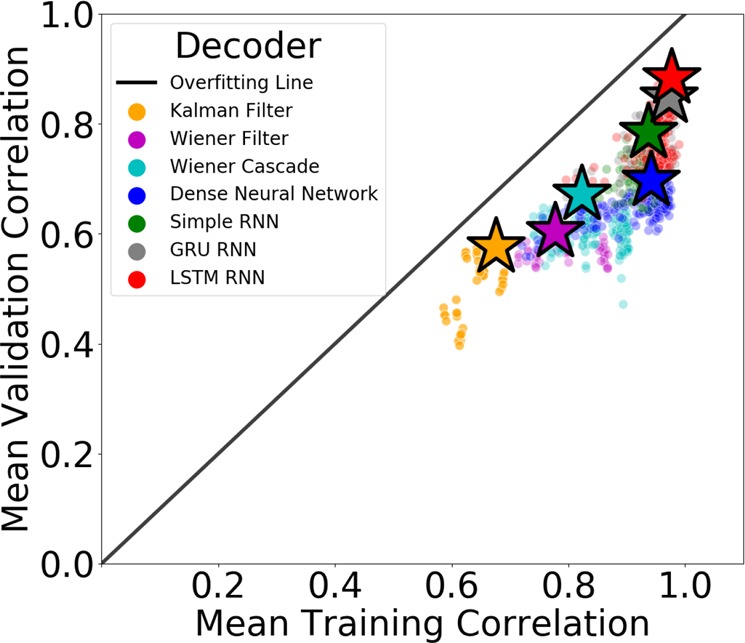
We evaluated the performance of seven different neural decoding algorithms (color-coded) over both the training data set (*x*-axis) and validation data set (*y*-axis). The top performing model for each algorithm is marked with a star. The distance from a given point to the overfitting line represents the degree to which the model overfit the training data.

We evaluated seven different neural decoding algorithms including the Kalman filter, Wiener filter, Wiener cascade, dense neural network (NN), simple recurrent NN (RNN), gated recurrent unit (GRU) RNN, and long short-term memory (LSTM) RNN (see “Methods” section). Each neural network consisted of a single hidden layer and an output layer. All models were trained on Google Cloud Platform n1-highmem-96 machines with Intel Skylake processors (see “Methods” section). We calculated a mean Pearson correlation between the target and predicted mel-spectrogram by calculating the correlation coefficient for each spectrogram band and averaging across bands^15^. We applied Fisher’s z-transform to correlation coefficients before averaging across spectrogram bands and before conducting statistical tests to impose additivity and to approximate a normal sampling distribution (see “Methods” section). Results are shown in Fig. 2.

We observed the Kalman filter (trained in 0.42 s) provided the lowest overall performance with the top model achieving a 0.57 mean validation correlation (MVC) and 0.68 mean training correlation (MTC). The top Wiener filter (trained in 2 s) performed similarly to the Kalman filter on the validation set (0.60 MVC) but showed an increased ability to fit the training set (0.78 MTC). A Wiener cascade (trained in 3 min and 33 s) of degree 4 beat the top Wiener filter with a MVC and MTC of 0.67 and 0.82, respectively.

A basic densely connected neural network (trained in 55 s) performed similarly to the top Wiener cascade decoder with a slightly improved MVC (0.69) and MTC (0.94). While the top simple RNN (trained in 2 min and 22 s) (0.94 MTC) decoder fit the training data as well as the dense neural network, it did generalize better to unseen data (0.78 MVC). Lastly, top GRU RNN (trained in 3 h and 46 min) (0.85 MVC, 0.97 MTC) and LSTM RNN (trained in 3 h and 37 min) (0.88 MVC, 0.98, MTC) decoders achieved similar performance with the LSTM RNN providing the best overall performance of all evaluated decoders on the validation set. The LSTM RNN also showed the highest robustness to overfitting with the top model performing only 8% worse on the validation set compared to the training set. For a comparison of how different neural network sizes affected performance, please see Supplementary Fig. 1. Note that neural network models were trained without the use of a GPU due to our heavy utilization of multiprocessing across hundreds of CPU cores (see “Methods” section). Future software iterations will enable multiprocessing on GPU-enabled systems.

To examine the statistical significance of these results, we performed an unbiased multiple comparisons statistical test followed by a post-hoc Tukey-type test^31^ using MVC values for the top-performing models (see “Methods” section). We found the LSTM RNN significantly outperformed all other decoding algorithms on the validation set (*p*-value $<$ 0.001) except for the GRU RNN (*p*-value $<$ 0.175). The GRU RNN significantly outperformed the simple RNN (*p*-value $<$ 0.017), and the simple RNN significantly outperformed the dense NN (*p*-value $<$ 0.017). Conversely, the dense NN did not significantly outperform the Wiener cascade (*p*-value $<$ 0.900) or the Wiener filter (*p*-value $<$ 0.086). These results indicate that recurrent neural networks (particularly, LSTM RNNs) provide a significant decoding improvement over traditional neural networks and other decoding methods on the performed audio reconstruction task. For a complete table of the decoding algorithm statistical significance results, please see Supplementary Table 1. For a comparison of reconstructed mel-spectrograms generated by each algorithm, please see Supplementary Fig. 2.

### Other decoding algorithm performance metrics

In addition to examining the mean correlation performance of neural decoding algorithms on the mel-spectrogram bands, we investigated how decoding algorithms recovered specific audio aspects including envelope, pitch, and loudness. We also quantified audio reconstruction intelligibility using the extended short-time objective intelligibility^23,32^ (ESTOI) and spectro-temporal modulation index^33^ (STMI) metrics. For a description of these metrics, please see “Methods” section.

**Fig. 3:**
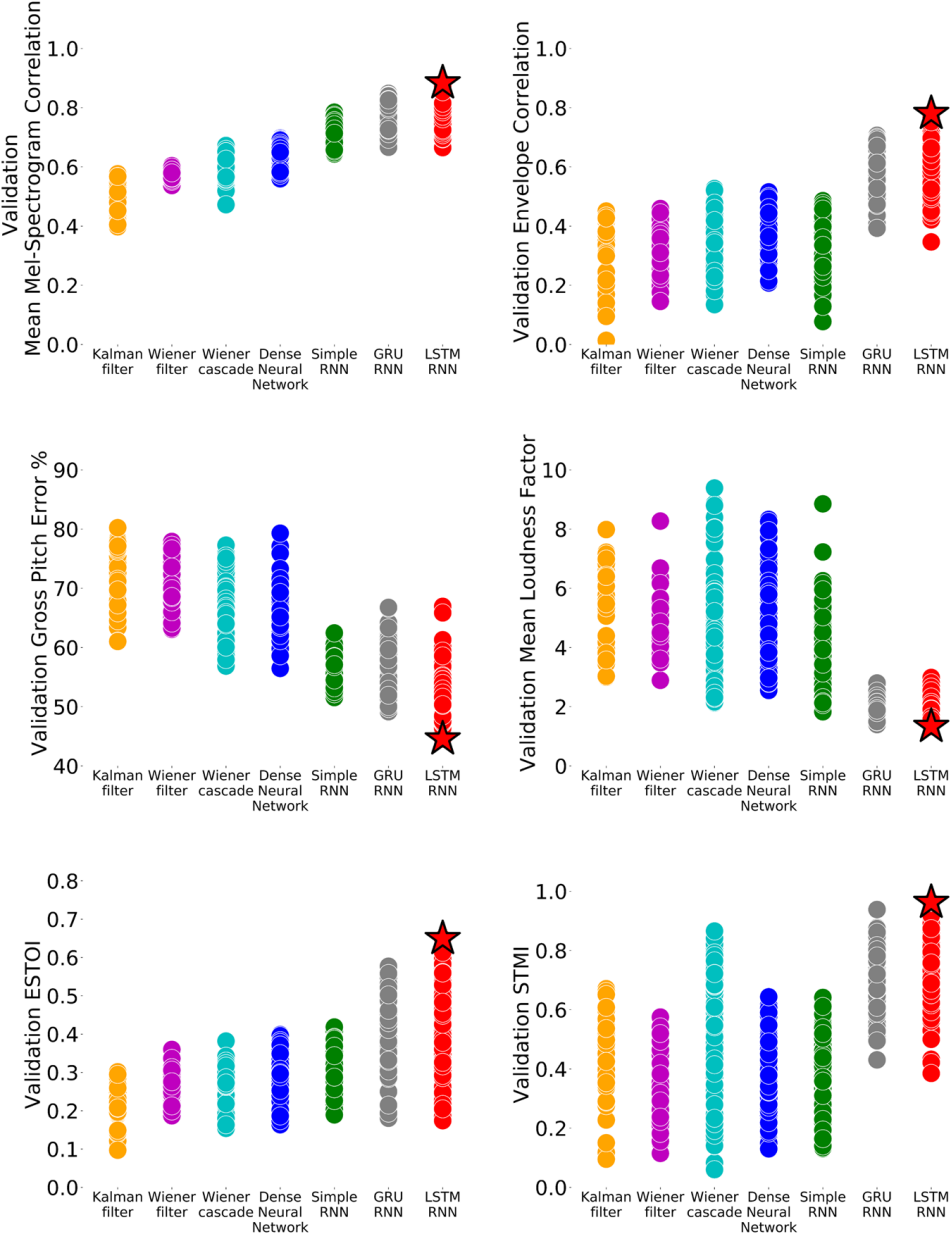
We compared the effectiveness of decoding algorithms at reconstructing various audio aspects and generating intelligible audio using six different performance metrics. The top-performing algorithm across all metrics was the LSTM RNN (red stars).

We found LSTM RNN decoders provided the best performance across all evaluated metrics (see Fig. 3). The LSTM RNN achieved the highest envelope correlation on the validation set compared to all algorithms (*p*-value $<$ 0.005) except for the GRU RNN (*p*-value $<$ 0.20). For gross pitch error, the LSTM performed similarly to the GRU RNN (*p*-value $<$ 0.29) and simple RNN (*p*-value $<$ 0.20) but outperformed all other algorithms (*p*-value $<$ 0.001). We observed a similar trend when evaluating the mean loudness factor as the LSTM RNN did not significantly outperform the GRU RNN (*p*-value $<$ 0.76) or simple RNN (*p*-value $<$ 0.11), but it did outperform all other algorithms (*p*-value $<$ 0.05). For intelligibility, the LSTM RNN performed similarly to the other neural network decoding algorithms on ESTOI including the dense NN (*p*-value $<$ 0.23), simple RNN (*p*-value $<$ 0.09), and GRU RNN (*p*-value $<$ 0.89) while performing significantly better than all other algorithms (*p*-value $<$ 0.008). Lastly, the LSTM RNN achieved a significantly higher STMI score than all other algorithms (*p*-value $<$ 0.001) except for the Wiener cascade (*p*-value $<$ 0.08) and GRU RNN (*p*-value $<$ 0.20).

### Neural feature extraction

To prepare neural features for decoding models, we bandpass filtered the raw neural data and calculated unsorted multiunit spike counts across all channels.

**Fig. 4:**
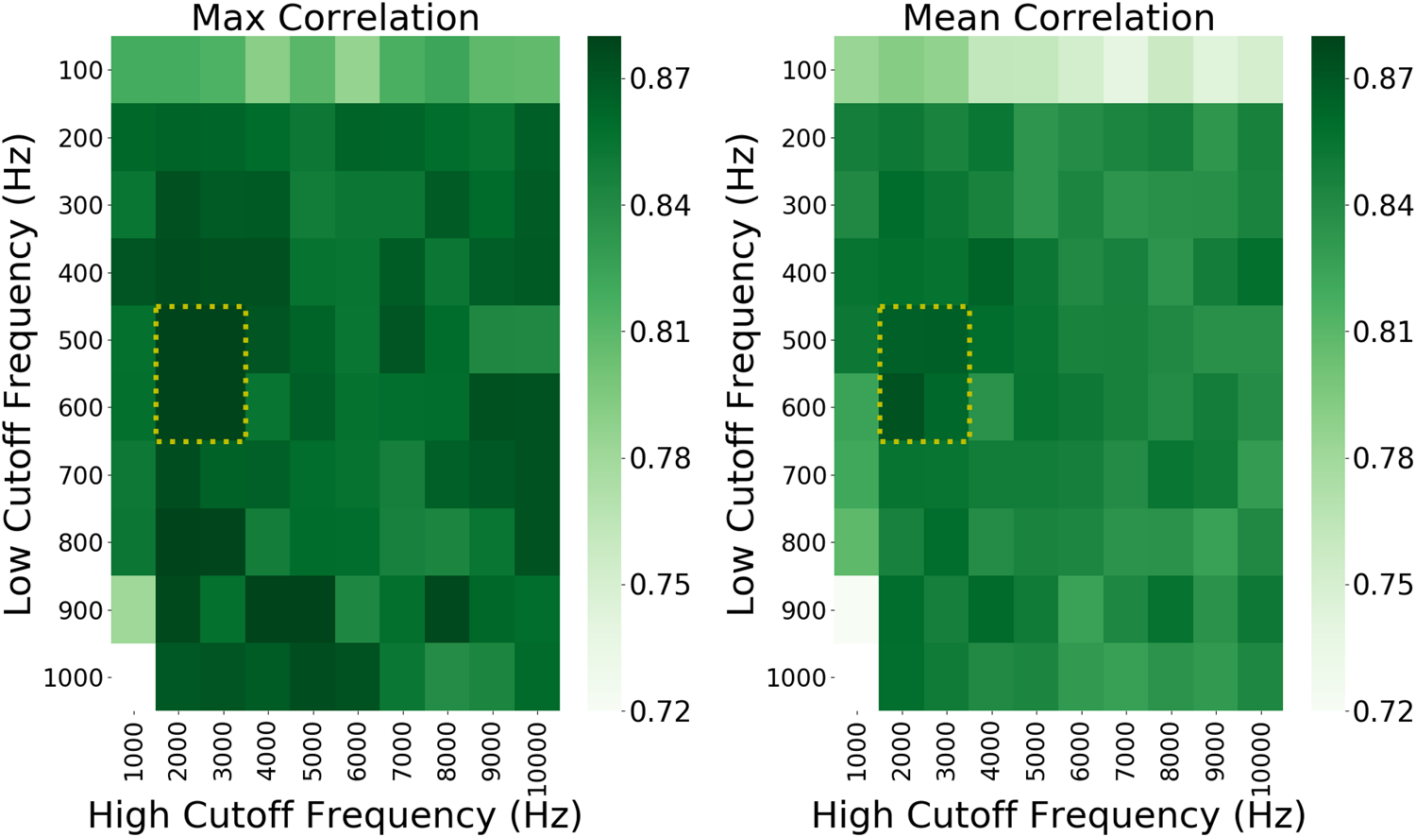
A performance heat-map of the grid-searched bandpass filter cutoff frequencies. We evaluated 4 LSTM RNN neural decoding models for 99 different bandpass filters. The left and right plots show max and mean performance, respectively, for the filters. We observed a marginal improvement in decoding performance when using a low cutoff of 500–600 Hz and a high cutoff of 2000–3000 Hz.

We first bandpass filtered the raw 30 kS/s neural data using a 2nd-order elliptic filter in preparation for threshold-based multiunit spike extraction. To explore the effect of the filter’s low and high cutoff frequencies, we performed a grid-search that evaluated 99 different bandpass filters. For each filter, four different LSTM RNN neural decoding models were evaluated. We found that using a low cutoff of 500–600 Hz and a high cutoff of 2000–3000 Hz (shown with yellow dotted lines) provided a marginal improvement in decoding performance (see Fig. 4).

We used multiunit (i.e., unsorted) spike counts as neural features for decoding models. After filtering, we calculated a noise level for each neural channel over the training set using median absolute deviation^34^. These noise levels were multiplied by a scalar “threshold factor” to set an independent spike threshold for every channel (same threshold factor used for all channels). We extracted negative threshold crossings from the filtered neural data (i.e., multiunit spikes) and binned them into spike counts over non-overlapping windows corresponding to the audio target sampling (see “Audio representation” section).

While we observed optimal values for the threshold factor, we found no clear pattern across decoding algorithms. The Wiener cascade was the most sensitive to threshold factor with a 9.1% difference between the most and least optimal values. GRU RNN decoders were the least sensitive to threshold factor with a 1.1% difference between the best and worst-performing values.

Except for the Kalman filter which processed a single input at a time, we used a window of sequential values for each feature as inputs to the decoding models. Each window was centered on the current prediction with its size controlled by a “feature window span” hyperparameter (length of the window not including the current value). We evaluated three different feature window spans (4, 8, and 16).

Both the GRU RNN and LSTM RNN showed higher performance with a larger feature window span (16); however, the other decoding algorithms achieved top performance with a smaller value (8). The LSTM RNN showed the most sensitivity to this hyperparameter with a 13.4% difference between a feature window span of 16 and 4. The Wiener filter was the least sensitive with a 3.1% difference between the best and worst-performing values. These results demonstrated the importance of determining the optimal feature window span when leveraging LSTM RNN neural decoding models.

Previous work has shown that increased neuronal firing rates correlate strongly with feature importance when using LSTM RNN models for neural decoding^35^. Therefore, to investigate the effect of channel count on decoding performance, we ordered neural channels according to the highest neural activity (i.e., highest counts of threshold crossings) over the training data set. For seven different channel counts (2, 4, 8, 16, 32, 64, and 96), we selected the top most active channels and built LSTM RNN decoding models using only the subselected channels.

**Fig. 5:**
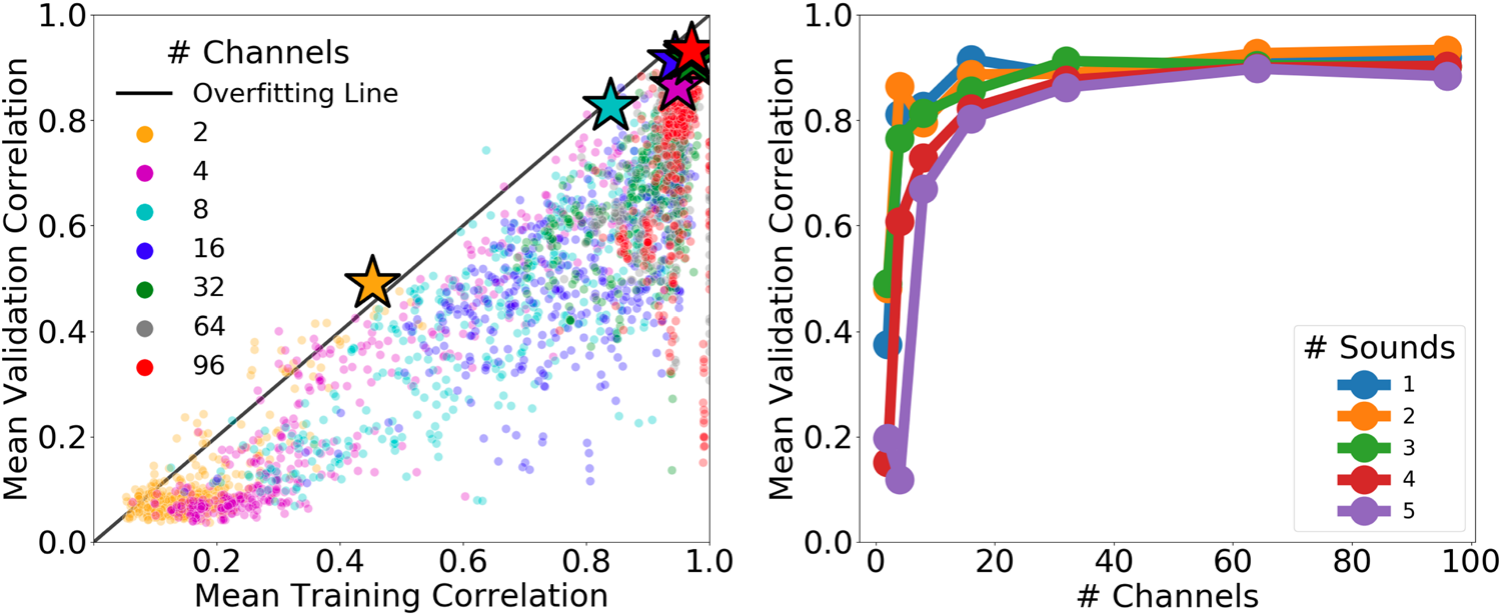
Every point represents an LSTM RNN neural decoder trained on audio data sets containing between 1 and 5 English words. In general, increased channel counts improved performance on more complex audio data sets. Note that the silver star (64 channels) is covered by the red star (96 channels) in the plot.

As shown in Fig. 5, we generally observed improvements in performance as channel count was increased. We found that selecting the 64 most active neural channels achieved the best performance on the validation set for an audio data set consisting of 5 English words.

### Audio representation

We converted raw recorded audio to a mel-frequency spectrogram^30^. We examined two hyperparameters for this process including the number of mel-bands in the representation and the hop size for the short-time fourier transform (STFT). We evaluated four different values for the number of mel-bands (32, 64, 128, and 256) and five different hop size values (10, 20, 30, 40, and 50 ms). To reconstruct audio, the mel-spectrogram was first inverted, and the Griffin-Lim algorithm^36^ was used to reconstruct phase information.

During preliminary studies, we observed increased decoder performance (mean validation correlations) when using fewer mel-bands and longer hop sizes. However, the process of performing mel-compression with these hyperparameter settings caused reconstructions to become unintelligible even when generated directly from the target mel-spectrograms (i.e., emulating a perfect neural decoding model). Therefore, by subjectively listening to the audio reconstructions generated from the target mel-spectrograms, we determined that a hop size of 40 ms and 128 mel-bands provided the best trade-off of decoder performance and result intelligibility.

To investigate the effect of audio complexity on decoding performance, we subselected different sets of sounds from the recorded audio data prior to building decoding models. Audio data sets containing 1 through 5 sounds were evaluated using the following English words: “tree”, “good”, “north”, “cricket”, and “program” (for macaque call results, see “Macaque call reconstruction” section). For each audio data set, we then varied the channel count (see “Neural feature extraction” section) across seven different values (2, 4, 8, 16, 32, 64, and 96).

As shown in Fig. 5, we found the optimal channel count varied with task complexity as increased channel counts generally improved performance on more complex audio data sets. However, we also observed decoders overfit the training data when the number of neural channels was too high relative to the audio complexity (i.e., number of sounds). Similar results have been observed when decoding high-dimensional arm movements using broadband population recordings of the primary motor cortex in NHPs^37^.

### Array location

**Fig. 6:**
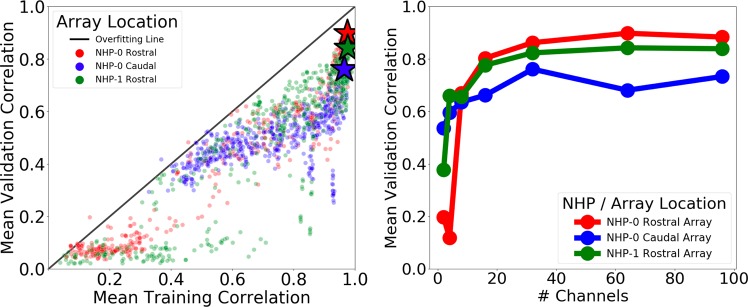
Intelligible audio reconstructions generated using three different arrays in two different NHPs in the RPB and CPB regions of the STG with RPB models achieving the highest performance on the validation set.

We implanted two NHPs (NHP-0 and NHP-1) with 96-channel MEAs in the rostral parabelt (RPB) and caudal parabelt (CPB) of the STG (see Fig. 1). We repeated the channel count experiment(see “Neural feature extraction” section) for three different arrays, including the NHP-0 RPB and CPB arrays and the NHP-1 RPB array (see Fig. 6). We successfully reconstructed intelligible audio from all three arrays with the RPB array in NHP-0 outperforming the other two arrays on the validation set (*p*-value $<$ 0.001). However, the successful reconstruction of intelligible audio from all three arrays suggests the neural representation of complex sounds is spatially distributed in the STG network. Future work will explore the benefit of synchronously recording from rostral and caudal arrays to enable decoding of more complex audio data sets.

### Macaque call reconstruction

In addition to the English words enumerated in Table 1, we investigated neural decoding models that successfully reconstructed macaque call audio from neural activity. One audio clip of a macaque call was randomly mixed in with the English word audio during the passive listening task.

The addition of the macaque call to the audio data set improved the achieved mean validation correlation from a 0.88 to a 0.95 despite increasing target audio complexity. This was due to the macaque call containing higher frequency spectrogram components compared to audio of the 5 English words (see “Audio preprocessing” section). By successfully learning to predict these higher frequency spectrogram bands, neural decoding models achieved a higher average correlation across the full spectrogram than when those same bands contained more noise. While adding the macaque call improved the average correlation scores, it also decreased the top ESTOI score from a 0.59 to a 0.56 on the validation set.

### Top performing neural decoder

Given an audio data set of 5 English words and decoding all 96 channels of neural data, the top performing neural decoder was a LSTM RNN (2048 recurrent units) model that achieved a 0.98 mean training correlation, 0.88 mean validation correlation, and 0.79 mean testing correlation. This model successfully reconstructed intelligible audio from RPB STG neural spiking activity with a validation ESTOI of 0.59 and a testing ESTOI of 0.54. Models were ranked by validation set mean mel-spectrogram correlation.

### Decoding larger audio sets and single trial audio

While the primary objective of this work was to enable future active listening NHP tasks that will consist of a limited number of English words ($\sim$3 to 5 words) and subsequent neural encoding work, we also performed a preliminary evaluation of the generalizability of the presented decoding methods on larger audio data sets and single trial audio presentations. We used the top performing neural decoder parameters (see “Top performing neural decoder” section) to train a model on a larger audio data set consisting of 17 English words. We then validated the resulting model on audio consisting of those 17 English words as well as 3 additional English words that were not included in the training set (i.e., single trial audio presentations). We found the resulting model successfully reconstructed the 17 training words (MVC of 0.90). The model also successfully reconstructed the simplest of the 3 single trial words (“two”) with performance decreasing as the word audio complexity increased (“cool” and “victory”) (see Table 2). These results suggest that the utilized decoding methods can generalize to single trial audio presentations given a sufficiently representative training set. Future work will further characterize the effect of training data audio complexity on decoder generalization.

**Table 2:** A decoding model trained on 17 English words successfully reconstructed a single trial English word not included in the training set (“two”).

Training words:	“program”, “macaque”, “laboratory”, “quality”, “good”, “tree”, “moo”, “apple”, “banana”, “cricket”, “zoo”, “fuel”, “reflection”, “north”, “sequence”, “window”, “error”
Validation words:	17 Training Words, “two”, “cool”, “victory”
Mean training correlation:	0.98
Mean validation correlation (all 20 words):	0.90
Mean validation correlation (“two”):	0.88
Mean validation correlation (“cool”):	0.65
Mean validation correlation (“victory”):	0.56

## Discussion

The work presented in this paper builds on prior foundational work in NHPs and human subjects in mapping and interpreting the role of the secondary auditory cortex by intracranial recording of neural responses to external auditory stimuli. In this paper, we hypothesized that the STG of the secondary auditory cortex is part of a powerful cortical computational network for processing sounds. In particular, we explored how complex sounds such as English language words were encoded even if unlikely to be cognitively recognized by the macaque subject.

We combined two sets of methods as part of our larger motivation to take steps towards human cortical and speech prostheses while leveraging the accessibility of the macaque model to chronic electrophysiology. First, the use of 96-channel intracortical MEAs was hypothesized to yield a new view of STG neural activity via direct recording of neural population (spiking) dynamics. To our knowledge, such multichannel implants have not been implemented in a macaque STG except for work that focused on the primary A1 and, to a lesser extent, the STG in this animal model^38^. Second, given the rapidly expanding use of deep learning techniques in neuroscience, we sought to create a suite of neural processing tools for high-speed parallel processing of neural decoding models. We also deployed methods developed for voice recognition and speech synthesis which are ubiquitous in modern consumer electronic applications. We demonstrated the reconstructed audio recordings successfully recovered the sounds presented to the NHP (see Supplementary Summary Movie^26^ and Correlation Movie^29^).

We performed an end-to-end neural decoding grid-search to explore the effects of signal properties, algorithms, and hyperparameters on reconstructing audio from full-broadband neural data recorded in STG. This computational experiment resulted in neural decoding models that successfully decoded neural activity into intelligible audio on the training, validation, and test data sets. Among the seven evaluated decoding algorithms, the Kalman filter and Wiener filter showed the worst performance as compared to the Wiener cascade and neural network decoders. This suggests that spiking activity in the parabelt region has a nonlinear relationship to the spectral content of sounds. This finding agrees with previous studies in which linear prediction models performed poorly in decoding STG activity^15,23,24^.

In this work, we found that recurrent neural networks outperformed other common neural decoding algorithms across six different metrics that measured audio reconstruction quality relative to target audio. These metrics evaluated reconstruction accuracy on mel-spectrogram bands, sound envelope, pitch, and loudness as well as two different intelligibility metrics. LSTM RNN decoders achieved the top scores across all six metrics. These findings are consistent with those of other studies in decoding neural activity recorded from the human motor cortex^39^, and recent work from our group has demonstrated the feasibility of using LSTM RNN decoders for real-time neural decoding^40^.

While LSTM RNN achieved the top performance on all evaluated metrics, we found no significant difference in performance between the LSTM RNN and GRU RNN across all performance metrics. Conversely, we observed a significant difference in performance from simple RNN decoders on three of the six metrics. These results indicated that the addition of gating units to the recurrent cells provided significant decoding improvements when reconstructing audio from STG neural activity. However, the addition of memory cells (present in LSTM RNN but not GRU RNN) did not significantly improve reconstruction performance. These results demonstrated that similar decoding performance was achieved whether the decoder used all available neural history (i.e., GRU RNN) or learned which history was most important (i.e., LSTM RNN). The similarity of the LSTM RNN and GRU RNN has also been observed when modeling polyphonic music and speech signals^41^.

In addition to the effect of decoding algorithm selection, we also showed that optimizing the neural frequency content via a bandpass filter prior to multiunit spike extraction provided a marginal decoding performance improvement. These results raise the question whether future auditory neural prostheses may achieve practically useful decoding performance while sampling at only a few kilohertz. We observed no consistent effect when varying the threshold factor used in extracting multiunit spike counts. When using an LSTM RNN neural decoder, longer windows of data and more LSTM nodes in the network improved decoding performance. In general, we found the complexity of the decoding task to affect the optimal channel count with increased channel counts improving performance on more complex audio data sets. However, decoders overfit the training data when the number of neural channels was too high relative to the audio complexity (i.e., number of sounds). Due to these results, we will continue to grid-search threshold factor, LSTM network size, and the number of utilized neural channels during future experiments.

While we observed some improvement in decoding performance from the MEAs implanted in the rostral parabelt STG compared to the array implanted in the caudal parabelt STG, this work does not fully answer the question whether location specificity of the MEA is critical within the overall cortical implant area, or whether reading out from the spatially extended cortical network in the STG is relatively agnostic to the precise location of the multichannel probe. Nonetheless, we plan to utilize information simultaneously recorded from two or more arrays in future work to test if this enables the decoding of increasingly complex audio (e.g., English sentence audio or sequences of macaque calls recorded in the home colony). We are developing an active listening task for future experiments that will allow the NHP subject to directly report different auditory percepts.

When comparing our results with previous efforts in reconstructing auditory representations from cortical activity, we note especially the work with human ECoG data^15,23^. On a numerical basis, our analysis achieved higher mean Pearson correlation and ESTOI values possibly due to the multichannel spike-based neural recordings and some advantage in such intracortical recordings compared to surface ECoG approaches. In our work with macaques, we also chose to restrict the auditory stimulus to relatively low complexity and short temporal duration (single words). Parenthetically, we note that the broader question of necessary required and optimal neural information versus task complexity has been approached theoretically^42^.

We also found that the audio representation and processing pipeline are critical to generating intelligible reconstructed audio from neural activity. Other studies have shown benefits from using deep learning to enable the audio representation/reconstruction^23^, and future work will explore these methods in place of mel-compression/Griffin-Lim algorithm. Given the goal of comparing different decoding models with our offline neural computational toolkit, we have not approached in this work the regime of real-time processing of neural signals such as required for a practical brain–computer interface. However, based on our previous work, the trained LSTM model could be implemented on a Field-Programmable Gate Array (FPGA) to achieve real-time latencies^40^. While we will continue to improve the performance of our neural decoding models, the presented results provide one starting point for future neural encoding work to “write in” neural information by patterned microstimulation^43,44^ to elicit naturalistic audio sensations. Such future work can leverage the results presented here in guiding steps towards the potential development of an auditory cortical prosthesis.

## Methods

### Research Subjects

This work included two male adult rhesus macaques. The animals had two penetrating MEAs (Blackrock Microsystems, LLC, Salt Lake City, UT) implanted in STG each providing 96 channels of broadband neural recordings. All research protocols were approved and monitored by Brown University Institutional Animal Care and Use Committee, and all research was performed in accordance with relevant NIH guidelines and regulations.

### Brain maps and surgery

The rostral and caudal parabelt regions of STG have been shown to play a role in auditory perception^3,45,46^. Those two parabelt areas are closely connected to the anterior lateral belt and medial lateral belt which show selectivity for the meaning of sound (“what”) and the location of the sound source (“where”), respectively^4^.

Our institutional experience with implanting MEAs in NHPs has suggested that there is a non-trivial failure rate for MEA titanium pedestals^47^. To enhance the longevity of the recording environment, we staged our surgical procedure in two steps. First, we created a custom planar titanium mesh designed to fit the curvature of the skull. This mesh was designed using a 3D-printed skull model of the target area (acquired by MRI and CT imaging) and was coated with hydroxyapatite to accelerate osseointegration. This mesh was initially implanted and affixed with multiple screws providing a greater surface area for osseointegration on the NHP’s skull. Post-surgical CT and MRI scans were combined to generate a 3D model showing the location of the mesh in relation to the skull and brain.

Several weeks after the first mesh implantation procedure, we devised a surgical technique to access the parabelt region. A bicoronal incision of the skin was performed. The incision was carried down to the level of the zygomatic arch on the left and the superior temporal line on the right. The amount of temporal muscle in the rhesus macaques prevented an inferior temporal craniotomy. To provide us with lower access on the skull base, we split the temporal muscle in the plane perpendicular to our incision (i.e., creating two partial thickness muscle layers) which were then retracted anteriorly and posteriorly. This allowed us to have sufficient inferior bony exposure to plan a craniotomy over the middle temporal gyrus and the Sylvian fissure.

**Fig. 7:**
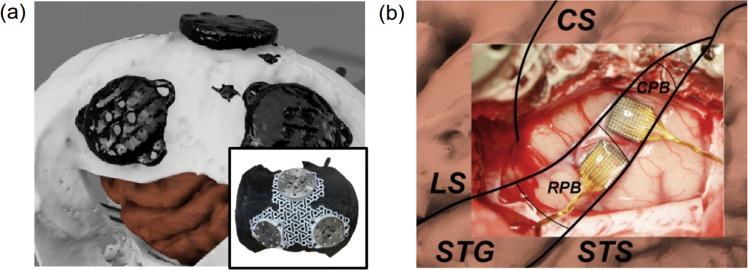
The anatomical structure of the cortex guided posistions of MEA on parabelt. **a** A 3D model of the skull, brain, and anchoring metal footplates (constructed by merging MRI and CT imaging). Also, a titanium mesh on a 3D-printed skull model. **b** Photo of the exposed area of the auditory cortex with labels added for relevant cortical structures (CS, central sulcus; LS, lateral sulcus; STG, superior temporal gyrus; STS, superior temporal sulcus; RPB, rostral parabelt; CPB, caudal parabelt).

The mesh, lateral sulcus, superior temporal sulcus, and central sulcus served as reference locations to guide the MEA insertion (see Fig. 7). MEA arrays were implanted with a pneumatic inserter^47^.

### NHP training and sound stimulus

We collected broadband neural data to characterize mesoscale auditory processing of STG during a passive listening task. While NHP subjects were restrained in a custom chair located in an echo suppression chamber using AlphaEnviro acoustic panels, complex sounds (English words and a macaque call) were presented through a focal loudspeaker. The subjects were trained to remain stable during the training session to minimize audio artifacts.

**Fig. 8:**
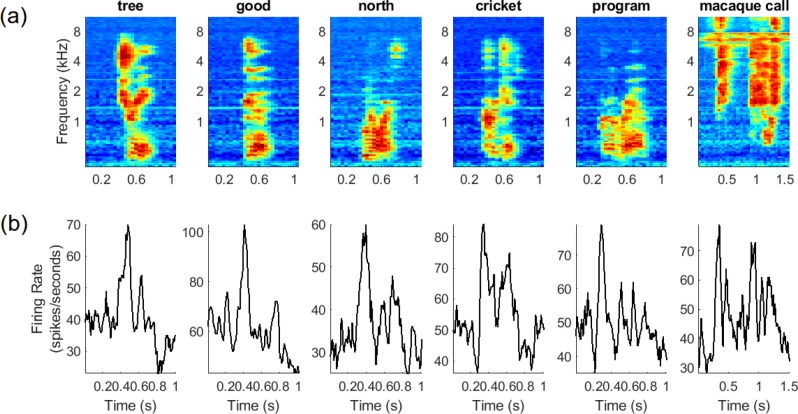
Neural data from the RPB array. **a** Mel-spectrograms (128 bands) of 5 English word sounds and one macaque call. **b** Histogram for multiunit spiking activity on a given recording channel. In all plots, the spectrogram hop size is 40 ms and the window size for firing rates is 10 ms. Note the different scales of the horizontal axes due to different sound lengths.

The passive listening task was controlled with a PC running MATLAB (Mathworks Inc., Natick, MA, Version 2018a). Animals were rewarded after every session using a treat reward. Within one session, 30 stimuli representations were played at ~1 s intervals. Data from 5 to 6 sessions in total were collected in one day. Computer synthesized English words were chosen to have different lengths (varying number of syllables) and distinct spectral contents (see Fig. 8).

In this work, a total of five different words were chosen and synthesized using MATLAB’s Microsoft Windows Speech API. Each sound was played 40–60 times with the word presentations pseudorandomly ordered. Trials that showed audio artifacts caused by NHP movement or environmental noise were manually rejected before processing.

### Intracortical multichannel neural recordings

We used MEAs from Blackrock Microsystems with an iridium oxide electrode tip coating that provided a mean electrode impedance of around 50 kOhm. Iridium oxide was chosen with future intracortical microstimulation in mind. The MEA electrode length was 1.5 and 0.4 mm pitch for high-density grid recording. Intracortical signals were streamed wirelessly at 30 kS/s per channel (12-bit precision, DC) using a CerePlex W wireless recording device^27,28^. Primary data acquisition was performed with a Digital Hub and Neural Signal Processor (NSP) (Blackrock Microsystems, Salt Lake City, UT). Audio was recorded at 30 kS/s synchronously with the neural data using a microphone connected to an NSP auxiliary analog input. The NSP broadcasted the data to a desktop computer over a local area network for long-term data storage using the Blackrock Central Software Suite. Importantly, the synchronous recording of neural and audio data by a single machine aligned the neural and audio data for offline neural decoding analysis.

### Neural processing toolkit and cloud infrastructure

We developed a neural processing toolkit^25^ (NPT) for performing large-scale distributed neural processing experiments in the cloud. The NPT is fully compatible with the dockex computational experiment infrastructure^48^ (Connexon Systems, Providence, RI). This enabled us to integrate software modules coded in different programming languages to implement processing pipelines. In total, the presented neural decoding analysis was composed of 26 NPT software modules coded in the Python, MATLAB, and C++ programming languages.

Dockex was used to launch, manage, and monitor experiments run on Google Cloud Platform (GCP) Compute Engine virtual machines (VMs). For this work, experiments were dispatched to a cluster of 10 VMs providing a total of 960 virtual CPU cores and over 6.5 terabytes of memory. In this work, GPUs were not used during the training or reconstruction since multiprocessing on GPUs was not yet supported by dockex; however, future experiments will leverage GPUs for accelerating deep learning. Machine learning experiment configuration files implemented the described grid-search by instantiating NPT modules with different combinations of parameters and defining dependencies between modules for synchronized execution and data passing. Dockex automatically extracted concurrency from these experiment configurations thereby scaling NPT execution across all available cluster resources. The presented neural processing experiments evaluated 12,779 unique neural decoding models.

### Neural preprocessing

We used multiunit spike counts (see Fig. 8) as neural features for the neural decoders. We leveraged the Combinato^49^ library to perform spike extraction via a threshold crossing technique. Raw neural data was first scaled to units of microvolts and filtered using a 2nd-order bandpass elliptic filter with grid-searched cutoff frequencies. A noise level (i.e., standard deviation) of each channel was estimated over the training set using the median absolute deviation to minimize the interference of spikes^34^. We set thresholds for each individual channel by multiplying the noise levels by a threshold factor. We detected negative threshold crossings on the full-broadband 30 kS/s data and then binned them into counts.

### Audio preprocessing

We manually labeled raw audio to designate the begin and end time indices for single sound trials. The audio data contained five different English words as well as a single macaque call. We subselected sounds to create target audio data sets for the neural decoders with varying complexity. The librosa audio analysis library^50^ was used to calculate the short-time Fourier transform spectrogram with an FFT window of 2048. This spectrogram was then compressed to its mel-scaled spectrogram to reduce its dimensionality to the number of mel-bands. These mel-bands served as the target data for the evaluated neural decoders. Audio files were recovered from the mel-bands by inverting the mel-spectrogram and using the Griffin-Lim algorithm^36^ to recover phase information. All targets were standardized to zero-mean using scikit-learn^51^ transformers fit on the training data.

### Decoding algorithms

We evaluated seven different neural decoding algorithms based on the KordingLab Neural_Decoding library^52^ including a Kalman filter, Wiener filter, Wiener cascade, dense neural network, simple recurrent RNN, GRU RNN, and LSTM RNN. Each neural network consisted of a single hidden layer and an output layer. Hidden units in the dense neural network and simple RNN used rectified linear unit activations^53^. The GRU RNN and LSTM RNN used tanh activations for hidden units. All output layers used linear activations, and no dropout^54^ was used. We used the adam optimization algorithm^55^ to train the dense neural network and RMSprop^56^ for all recurrent neural neural networks. We sequentially split the data set ($\sim$40 trials per sound per array sampled in 40 ms bins) into training, validation, and testing sets composed of 80%, 10%, and 10% of the data, respectively. We trained all neural networks using early stopping^57^ with a maximum of 2048 training epochs and a patience of 5 epochs. Mean-squared error was used as the monitored loss metric.

### Decoding model comparison metrics

We utilized six different performance metrics for assessing and comparing the performance of neural decoding models. We selected metrics to investigate the reconstruction of different audio aspects and to quantify reconstruction speech intelligibility.

For each mel-spectrogram frequency band, Pearson’s correlation coefficient was calculated between the target and predicted values. Fisher’s z-transform was applied to impose additivity on the correlation coefficients before taking the mean of the transformed correlations across frequency bands. The inverse z-transform was applied to the resulting mean value to calculate the reported correlation values. This process followed previous auditory cortex decoding work^15^ and provided a spectral accuracy metric domain for audio reconstructions.

The temporal envelope of human speech includes information in the 2–50 Hz range and provides segmental cues to manner of articulation, voicing, vowel identification as well as prosodic cues^58^. We found the temporal envelope of the target and reconstructed audio by calculating the magnitude of the Hilbert transform and low-pass filtering the results at 50 Hz^59^. We then calculated envelope correlation by finding the Pearson correlation between the target and reconstructed envelopes.

Gross pitch error (GPE) represents the proportion of frames where the relative pitch error is higher than 20%^60^. Pitch of the target ($F0$) and reconstructed audio ($\hat{F0}$) was found with the Normalized Correlation Function method^61^ through the MATLAB pitch function^62^. This resulted in an estimation of the momentary fundamental frequency for the target and reconstructed audio. GPE was then calculated using the following formula:

1 $${\mathrm{{GPE}}}=\frac{{N}_{error}}{N}* 100 \%$$

where $N$ is the total number of frames, and ${N}_{error}$ is the number of frames for which $\left|F0-\hat{F0}\right|> F0* p$ with $p=0.2$.

Momentary loudness for the target and reconstructed audio was calculated in accordance with the EBU R 128 and ITU-R BS.1770-4 standards using the MATLAB loudnessMeter object^63^. This resulted in loudness values with units of loudness units relative to full scale (LUFS) where 1 LUFS = 1 dB. For the purpose of comparing overall loudness reconstruction accuracy across decoding algorithms, we calculated the absolute value of the momentary decibel difference between the target loudness ($l$) and reconstructed loudness ($\hat{l}$). By utilizing the absolute value of differences between the target and reconstructed loudness, this process equally penalized reconstructions that were too soft or too loud (analogous to Mean Absolute Percent Error). We then calculated a momentary loudness factor (${\mathrm{{LF}}}$) assuming a +10 dB change in loudness corresponds to a doubling of perceived loudness^64^ using the following equation:

2 $${\mathrm{{LF}}}={2}^{\frac{\left|{\bf{l}}-\hat{{\bf{l}}}\right|}{10}}$$

We reported the mean of the momentary loudness factor over time as the overall loudness accuracy metric where values closer to 1 represent better loudness reconstruction.

The ESTOI algorithm estimates the average intelligibility of noisy audio samples across a group of normal-hearing listeners^32^. ESTOI calculates intermediate intelligibility scores over 384 ms time windows before averaging the intermediate scores over time to calculate a final intelligibility score. Intermediate intelligibility scores are found by passing the target (i.e., clean) and reconstructed (i.e., noisy) audio signals through a one third octave filter bank to model signal transduction in the cochlear inner hair cells. The subband temporal envelopes for both signals are then calculated before performing normalization across the rows and columns of the spectrogram matrices. The intermediate intelligibility is defined as the signed length of the orthogonal projection of the noisy normalized vector onto the clean normalized vector.

STMI is a speech intelligibility metric which quantifies the joint degradation of spectral and temporal modulations resulting from noise^33^. STMI utilizes a model of the mammalian auditory system by first generating a neural representation of the audio (auditory spectrogram) and then processing that neural representation with a bank of modulation selective filters to estimate the spectral and temporal modulation content. Here, we utilized the STMI specific to speech samples ($\mathrm{STM}\mathrm{I}^{\mathrm{{T}}}$) which averages the spectro-temporal modulation content across time and calculates the index using the following formula:

3 $$\mathrm{STM}\mathrm{I}^{\mathrm{{T}}}=1-\frac{{\left\Vert {\bf{T}}-{\bf{N}}\right\Vert }^{{\rm{2}}}}{{\left\Vert {\bf{T}}\right\Vert }^{{\rm{2}}}}$$

where $T$ is the true audio (i.e., target) and $N$ is the noisy audio (i.e., reconstructed). This approach captures the joint effect of spectral and temporal noise which other intelligibilty metrics (e.g. Speech Transmission Index) fail to capture.

### Statistics and reproducibility

For testing the significance of differences in mel-spectrogram mean Pearson correlation, we followed a procedure described by Paul (1988)^31^. We first applied Fisher’s z-transform to the correlation values to normalize the underlying distributions of the correlation coefficients and to stabilize the variances of these distributions. We then tested a multisample null hypothesis using an unbiased one-tailed test by calculating a chi-square value using the following equation:

4 $${\chi }_{P}^{{\rm{2}}}=\sum _{i=1}^{k}\frac{{n}_{i}* {({r}_{i}-{r}_{w})}^{{\rm{2}}}}{{(1-{r}_{i}{r}_{w})}^{{\rm{2}}}}$$

with $k-1$ degrees of freedom where ${n}_{i}$ is the population sample size, ${r}_{i}$ is the population correlation coefficient, and ${r}_{w}$ is the common correlation coefficient. The resulting values rejected the null hypothesis, and we then applied a post-hoc Tukey-type test to perform multiple comparisons across decoding algorithms and hyperparameters.

For all other performance metrics, we first split the reconstructed validation audio into non-overlapping blocks of 2 s in length. A given performance metric was then calculated for each block, and a Friedman test was performed to test the multisample null hypothesis. For all evaluated metrics, this process rejected the null hypothesis, and we then applied Conover post-hoc multiple comparisons tests to calculate the significance of differences between decoding algorithms. We utilized the STAC^65^ Python Library to perform the Friedman tests and the scikit-posthocs^66^ Python library to perform the Conover tests.

Data was collected from 2 NHPs using a total of three different arrays. For each array, we utilized $\sim$40 trials of each sound (i.e., English words and macaque call) for analysis. These trials were collected over multiple sessions.

### Reporting summary

Further information on research design is available in the Nature Research Reporting Summary linked to this article.

## Supplementary information

Supplementary Material

Description of Additional Supplementary Files

Supplementary Movie 1

Supplementary Movie 2

Reporting Summary

## Data Availability

Neural processing code used in this study is available online^25^.

## Data availability

The data that supports the findings of this study are available upon request from the corresponding authors [C.H., J.L., and A.N.].
