# Supplementary material for: Decoding speech from spike-based neural population recordings in secondary auditory cortex of non-human primates
Source: Commun Biol. 2019 Dec 11;2:466. doi: 10.1038/s42003-019-0707-9 (PMC6906475; doi:10.1038/s42003-019-0707-9)
Supplement: Supplementary file 1 — Supplementary Material [file 42003_2019_707_MOESM1_ESM.pdf]

Supplementary Figures

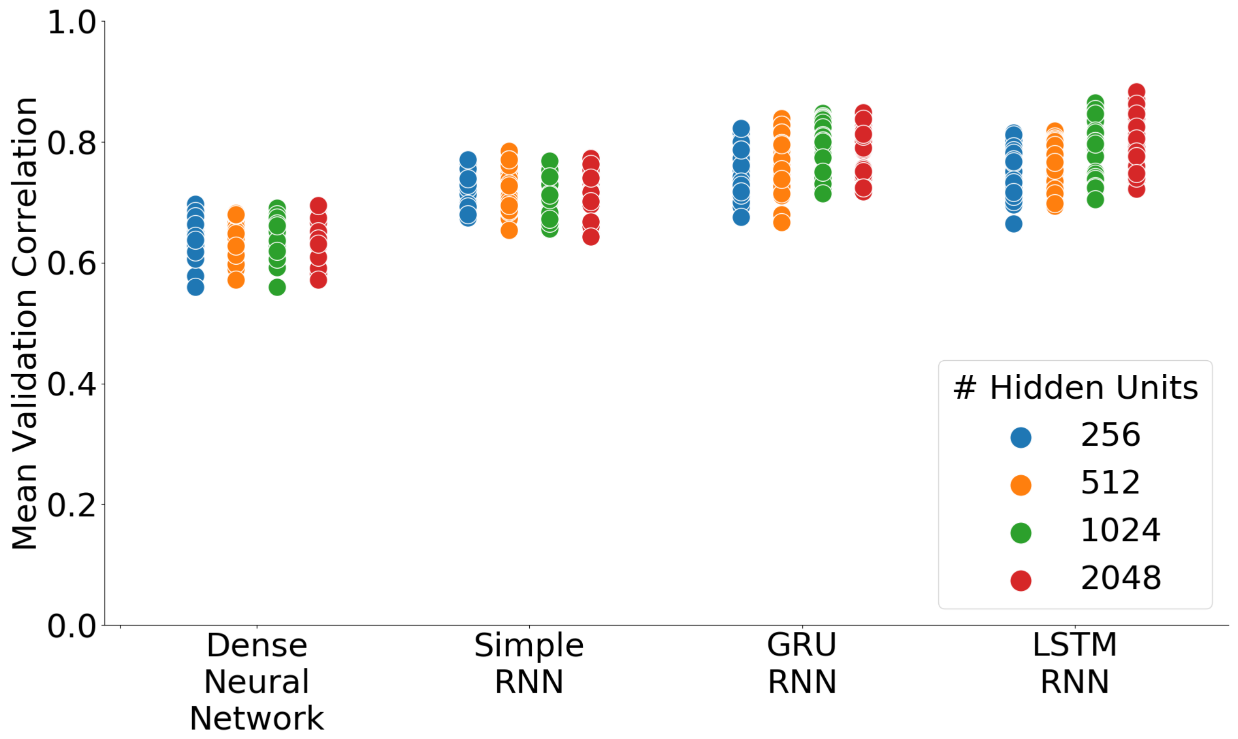

Supplementary Figure 1: The optimal number of hidden units for neural network decoders varied across network types. The top-performing LSTM RNN algorithm achieved its highest decoding performance with a larger number of hidden units (2048).

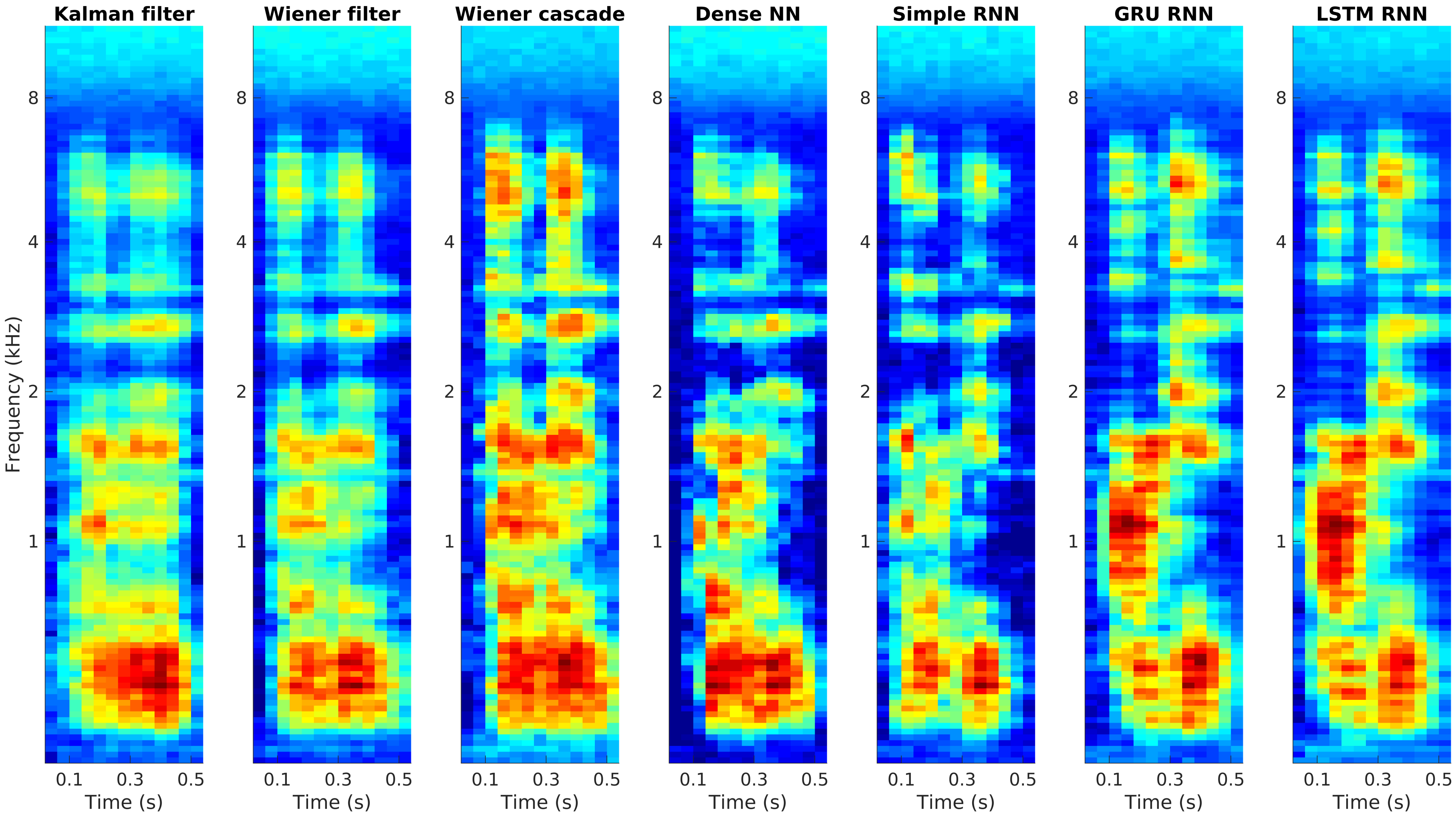

Supplementary Figure 2: Each mel-spectrogram shows a reconstruction of the word ”cricket” generated by the top-performing model for each algorithm.

Supplementary Tables

Supplementary Table 1: The results of the decoding algorithm multiple comparisons Tukey-type test.

B	A	Correlation B	Correlation A	Adjusted p-value	Result
LSTMRNN	GRURNN	0.88	0.85	0.175	Correlations NOT significantly different
LSTMRNN	SimpleRNN	0.88	0.78	0.001	Correlation B significantly greater than Correlation A
LSTMRNN	DenseNeuralNetwork	0.88	0.69	0.001	Correlation B significantly greater than Correlation A
LSTMRNN	WienerCascade	0.88	0.67	0.001	Correlation B significantly greater than Correlation A
LSTMRNN	WienerFilter	0.88	0.60	0.001	Correlation B significantly greater than Correlation A
LSTMRNN	KalmanFilter	0.88	0.57	0.001	Correlation B significantly greater than Correlation A
GRURNN	SimpleRNN	0.85	0.78	0.017	Correlation B significantly greater than Correlation A
GRURNN	DenseNeuralNetwork	0.85	0.69	0.001	Correlation B significantly greater than Correlation A
GRURNN	WienerCascade	0.85	0.67	0.001	Correlation B significantly greater than Correlation A
GRURNN	WienerFilter	0.85	0.60	0.001	Correlation B significantly greater than Correlation A
GRURNN	KalmanFilter	0.85	0.57	0.001	Correlation B significantly greater than Correlation A
SimpleRNN	DenseNeuralNetwork	0.78	0.69	0.017	Correlation B significantly greater than Correlation A
SimpleRNN	WienerCascade	0.78	0.67	0.001	Correlation B significantly greater than Correlation A
SimpleRNN	WienerFilter	0.78	0.60	0.001	Correlation B significantly greater than Correlation A
SimpleRNN	KalmanFilter	0.78	0.57	0.001	Correlation B significantly greater than Correlation A
DenseNeuralNetwork	WienerCascade	0.69	0.67	0.900	Correlations NOT significantly different
DenseNeuralNetwork	WienerFilter	0.69	0.60	0.086	Correlations NOT significantly different
DenseNeuralNetwork	KalmanFilter	0.69	0.57	0.006	Correlation B significantly greater than Correlation A
WienerCascade	WienerFilter	0.67	0.60	0.437	Correlations NOT significantly different
WienerCascade	KalmanFilter	0.67	0.57	0.076	Correlations NOT significantly different
WienerFilter	KalmanFilter	0.60	0.57	0.900	Correlations NOT significantly different
