# Supplementary material for: Decoding speech from spike-based neural population recordings in secondary auditory cortex of non-human primates
Source: Commun Biol. 2019 Dec 11;2:466. doi: 10.1038/s42003-019-0707-9 (PMC6906475; doi:10.1038/s42003-019-0707-9)
Supplement: Supplementary file 2 — Description of Additional Supplementary Files [file 42003_2019_707_MOESM2_ESM.pdf]

Description of Additional Supplementary Files

File Name: **Supplementary Movie 1**
Description: This video summarizes the presented findings and demonstrates audio reconstructions generated by the top-performing neural decoding model for both the validation set and test set.

File Name: **Supplementary Movie 2**
Description: During our analysis, we primarily used mean Pearson correlation between the target and predicted audio mel-spectrogram bands as a performance metric for neural decoding models. In this video, we present examples that demonstrate various reconstructions and their corresponding correlation scores. This video aims to provide subjective context to the reader regarding the intelligibility of our experimental results.
