# Supplementary material for: Decoding speech from spike-based neural population recordings in secondary auditory cortex of non-human primates
Source: Commun Biol. 2019 Dec 11;2:466. doi: 10.1038/s42003-019-0707-9 (PMC6906475; doi:10.1038/s42003-019-0707-9)
Supplement: Supplementary file 5 — Reporting Summary [file 42003_2019_707_MOESM5_ESM.pdf]

nature research

Corresponding author(s): Christopher Heelan, Jihun Lee, Arto V. Nurmikko

Last updated by author(s): Nov 4, 2019

# Reporting Summary

Nature Research wishes to improve the reproducibility of the work that we publish. This form provides structure for consistency and transparency in reporting. For further information on Nature Research policies, see Authors & Referees and the Editorial Policy Checklist.

## Statistics

For all statistical analyses, confirm that the following items are present in the figure legend, table legend, main text, or Methods section.

| n/a | Confirmed | |
|---|---|---|
| ☐ | ☒ | The exact sample size (*n*) for each experimental group/condition, given as a discrete number and unit of measurement |
| ☐ | ☒ | A statement on whether measurements were taken from distinct samples or whether the same sample was measured repeatedly |
| ☐ | ☒ | The statistical test(s) used AND whether they are one- or two-sided<br>*Only common tests should be described solely by name; describe more complex techniques in the Methods section.* |
| ☐ | ☒ | A description of all covariates tested |
| ☐ | ☒ | A description of any assumptions or corrections, such as tests of normality and adjustment for multiple comparisons |
| ☐ | ☒ | A full description of the statistical parameters including central tendency (e.g. means) or other basic estimates (e.g. regression coefficient) AND variation (e.g. standard deviation) or associated estimates of uncertainty (e.g. confidence intervals) |
| ☐ | ☒ | For null hypothesis testing, the test statistic (e.g. *F*, *t*, *r*) with confidence intervals, effect sizes, degrees of freedom and *P* value noted<br>*Give P values as exact values whenever suitable.* |
| ☒ | ☐ | For Bayesian analysis, information on the choice of priors and Markov chain Monte Carlo settings |
| ☒ | ☐ | For hierarchical and complex designs, identification of the appropriate level for tests and full reporting of outcomes |
| ☐ | ☒ | Estimates of effect sizes (e.g. Cohen's *d*, Pearson's *r*), indicating how they were calculated |

*Our web collection on statistics for biologists contains articles on many of the points above.*

## Software and code

Policy information about availability of computer code

| | |
|---|---|
| Data collection | Blackrock Microsystems Central Software Suite v7 |
| Data analysis | Mathworks MATLAB 2018a, Connexon Systems dockex v0.1, Nurmikko Lab Neural Processing Toolkit v0.1 (custom) |

For manuscripts utilizing custom algorithms or software that are central to the research but not yet described in published literature, software must be made available to editors/reviewers. We strongly encourage code deposition in a community repository (e.g. GitHub). See the Nature Research guidelines for submitting code & software for further information.

## Data

Policy information about availability of data

All manuscripts must include a data availability statement. This statement should provide the following information, where applicable:

- Accession codes, unique identifiers, or web links for publicly available datasets
- A list of figures that have associated raw data
- A description of any restrictions on data availability

The data that supports the findings of this study are available upon request from the corresponding authors [C.H., J.L., A.N.].

# Field-specific reporting

Please select the one below that is the best fit for your research. If you are not sure, read the appropriate sections before making your selection.

☒ Life sciences ☐ Behavioural & social sciences ☐ Ecological, evolutionary & environmental sciences

For a reference copy of the document with all sections, see nature.com/documents/nr-reporting-summary-flat.pdf

# Life sciences study design

All studies must disclose on these points even when the disclosure is negative.

| | |
|---|---|
| Sample size | Two non-human primates (NHPs) were implanted with microelectrode arrays in the superior temporal gyrus. We collected and analyzed data from a total of 3 arrays. |
| Data exclusions | Any sections of data that contained audio noise recorded by the microphone (e.g. experimenters talking, animals moving) were discarded to ensure the data set consisted of only the desired audio (English words and NHP calls). |
| Replication | Machine learning experiments were performed on data sets collected from 3 different microelectrode arrays implanted in the superior temporal gyrus. Intelligible audio was successfully reconstructed from all neural data across all arrays. |
| Randomization | Audio samples (English words and NHP calls) played through the speaker were randomly ordered. |
| Blinding | Blinding was not necessary for this work as there is no separate control and experimental group. |

# Reporting for specific materials, systems and methods

We require information from authors about some types of materials, experimental systems and methods used in many studies. Here, indicate whether each material, system or method listed is relevant to your study. If you are not sure if a list item applies to your research, read the appropriate section before selecting a response.

## Materials & experimental systems

| n/a | Involved in the study | |
|---|---|---|
| ☒ | ☐ | Antibodies |
| ☒ | ☐ | Eukaryotic cell lines |
| ☒ | ☐ | Palaeontology |
| ☐ | ☒ | Animals and other organisms |
| ☒ | ☐ | Human research participants |
| ☒ | ☐ | Clinical data |

## Methods

| n/a | Involved in the study | |
|---|---|---|
| ☒ | ☐ | ChIP-seq |
| ☒ | ☐ | Flow cytometry |
| ☒ | ☐ | MRI-based neuroimaging |

## Animals and other organisms

Policy information about studies involving animals; ARRIVE guidelines recommended for reporting animal research

| | |
|---|---|
| Laboratory animals | This study included two male adult rhesus macaques. |
| Wild animals | This study did not involve wild animals. |
| Field-collected samples | This study did  not involve samples collected from the field. |
| Ethics oversight | All research protocols were approved and monitored by Brown University Institutional Animal Care and Use Committee, and all research was performed in accordance with relevant NIH guidelines and regulations. |

Note that full information on the approval of the study protocol must also be provided in the manuscript.
